# Mechanistic Insights into Quorum Quenching-Mediated Control of EPS and Biofilm Formation in Submerged MBR

**DOI:** 10.3390/molecules31061022

**Published:** 2026-03-19

**Authors:** Noman Sohail, Marion Martienssen

**Affiliations:** Chair of Biotechnology of Water Treatment, Brandenburg University of Technology Cottbus-Senftenberg, Siemens-Halske-Ring 8, 03046 Cottbus, Germany; marion.martienssen@b-tu.de

**Keywords:** membrane bioreactor (MBR), polytetrafluoroethylene (PTFE), polysulfones (PSs), extracellular polymeric substances (EPSs)

## Abstract

Quorum quenching (QQ) is a promising biological approach that has the potential to control membrane biofouling. However, the implementation of the QQ membrane bioreactor still requires a more systematic and comprehensive understanding, including the selection of membrane materials, the determination of the optimal QQ bacterial dosage, and the use of appropriate media for the immobilization of QQ bacteria, all of which are important to ensure long-term operation. The present study investigated the impact of QQ bacteria on biofilm formation across different polymeric membranes. These include flat sheet membranes, Polytetrafluoroethylene (PTFE), Polysulfones (PSs), and hollow-fibre polyvinylidene difluoride (PVDF) membranes. It also evaluated biofilm development, membrane filtration performance, extracellular polymeric substance (EPS) production, and sludge floc properties, which were characterized using fluorescence microscopy. The results revealed that QQ intervention markedly suppressed quorum sensing (QS), leading to a pronounced, dose-dependent reduction in biofilm thickness, membrane fouling, EPS production and sludge floc size. Biofilm thickness was reduced by 63.5% on PTFE and 55.4% on PS membranes, accompanied by a notable reduction in EPS protein and polysaccharides, thereby weakening the biofilm formation and enhancing membrane filterability. Therefore, the permeability performance of the PVDF membrane improved by 338.2%. Furthermore, sludge settleability was enhanced, and floc size was reduced, resulting in the mitigation of biofilm formation without impacting pollutant degradation. These findings elucidate the material-dependent and dose-responsive mechanism by which QQ regulates EPS synthesis and biofilm formation in MBR.

## 1. Introduction

A membrane bioreactor (MBR) is an advanced wastewater treatment technology that integrates a biological process with membrane filtration [1]. An MBR produces a high effluent quality with better removal efficiency of organic compounds and ammonia. It also eliminates the need for a sedimentation basin and overcomes the sludge settling problem [2]. This allows MBRs to operate at a longer solid retention time (SRT) without facing the sludge settling problem and inefficient solid-liquid separation, which was typically a major challenge in the conventional activated sludge process (CASP), thereby achieving better biodegradation and reduced biomass production [3]. Furthermore, it is a single-step process in which the concentration of mixed liquor suspended solids (MLSS) can be easily maintained at 8 to 12 g L^−1^, which helps to achieve high removal efficiency, even at a minimum hydraulic retention time (HRT), a level that is difficult to achieve in the CASP [4]. It also enhances the nitrification/denitrification process, with a shorter HRT and a smaller footprint [5]. Despite the unique advantages of MBR technology, there is one major drawback, membrane biofouling, which is a major concern for its large-scale application [6].

Membrane biofouling is associated with the undesired accumulation of microorganisms on the membrane surface [7]. The process of attachment is facilitated by the secretion of extracellular polymeric substances (EPSs) by microorganisms, followed by scaling and microbial colonization on the membrane, which leads to a reduction in treated wastewater [8]. Important contributors include EPS proteins and polysaccharides, soluble microbial products (SMPs), and humic substances, which form a strong biocake layer on the membrane surface [9,10]. As a consequence, membrane fouling leads to an increase in transmembrane pressure (TMP) under constant-flux operation, due to blockage of the pores from the inside and outside and the formation of a cake layer on the surface of the membrane [11]. Biofouling is monitored through TMP, because blockage of the membrane pores increases resistance to permeate flow and ultimately leads to a rise in TMP [12]. Membrane biofouling progresses in four steps, beginning with the initial attachment of microbial cells to the membrane surface, followed by the secretion of microbial products. Subsequently, EPSs are released to form a cohesive matrix that promotes biofilm maturation. Finally, the microbial community grows and proliferates, eventually leading to cell detachment and biofilm dispersal [13].

Biofilm formation varies across polymeric membranes and is significantly influenced by their specific physicochemical properties. Factors such as surface roughness, surface charge, pore size, hydrophobicity/hydrophilicity, porosity, and module design all play critical roles in primary microbial adhesion. Polyvinylidene fluoride (PVDF), polytetrafluoroethylene (PTFE), and polystyrene (PS) have been reported as common membrane materials in MBR systems [5,14]. Surface energy and surface charge also influence electrostatic interactions and EPS deposition, which contribute to the structural stability of the biofilm. Surface roughness further enhances bacterial colonization by providing protected niches for microbial accumulation compared to smoother surfaces. Therefore, variations in membrane material properties can substantially influence biofilm formation dynamics and fouling behaviour [15].

Quorum sensing (QS) is a bacterial cell-to-cell communication system that uses signal molecules as autoinducers (AIs) for communication [16]. Acyl homoserine lactones (AHLs) are one of the main types of AIs used by Gram-negative bacteria to adopt different types of social behaviours, including biofilm formation. It has been previously reported that Gram-negative bacteria have a higher abundance than Gram-positive bacteria in MBRs. Therefore, AHLs are considered one of the main AIs responsible for biofilm formation [17]. QS regulates bacterial gene expression in response to population density. When the concentration of QS molecules reaches a certain level, they bind to specific protein receptors and activate the transcription of genes involved in biofilm formation [18,19]. Several studies have demonstrated the correlation between QS and biofouling, which is a biologically driven process caused by bacterial activity. Accordingly, these studies have revealed that the biological solution known as quorum quenching (QQ) can disrupt QS signalling pathways and thereby mitigate membrane biofouling [20,21,22].

The QQ approach is employed to disrupt QS communication effectively and thereby mitigate biofouling [23]. This mechanism degrades signal molecules (AHLs) by releasing QQ enzymes such as lactonase and acylase [24,25]. Recent studies have shown that the entrapment of QQ bacteria into beads, such as *Rhodococcus* sp. BH4, *Pseudomonas* sp. 1A1 and *Brucella* sp. ZJ1, reduces EPS content and membrane fouling by disrupting QS signal molecules, thereby increasing the operation duration, minimizing TMP and prolonging membrane lifespan [26,27]. Immobilization of QQ bacteria is essential for long-term interference in the QS pathway. Researchers have used various types of media, including vessels, beads, cylinders, hollow cylinders and sheets, to entrap the QQ bacteria and assess their efficiency and activity in different media [28]. The surface area of the QQ media plays an important role in enhancing the QQ activity [29]. In order to protect QQ bacteria from harsh environmental conditions, as well as bacterial competitors from the MBR microbial community, and to prevent the loss of QQ cells during sludge removal, they are preferably entrapped in QQ beads or vessels [30]. Therefore, PVA-based bead media are considered more stable and cost-effective for the long-term operation of cell entrapment [31].

This study investigates the potential of QQ activity as a biofouling control strategy by assessing its impact on biofilm formation across different polymeric membranes. This was investigated by examining biofilm thickness, membrane fouling and EPS production, with a particular emphasis on proteins and polysaccharides, because they play an important role in biofilm adhesion. The study examined a hollow-fibre polyvinylidene difluoride (PVDF) membrane and flat-sheet polytetrafluoroethylene (PTFE) and polysulfone (PS) membranes. PVDF membranes were used for filtration, while PTFE and PS membranes were used to assess biofilm formation, microbial-material interaction and EPS content. To enhance the practical application of *Rhodococcus* sp. BH4 was encapsulated in polyvinyl alcohol and sodium alginate (PVA-SA) beads. The effectiveness of the QQ strain was assessed to measure the mitigation of biofilm thickness and membrane fouling. This study provides insight into the role of QQ-based strategies using different membrane materials, providing an advanced understanding of QQ’s applicability for biofouling control in MBR.

## 2. Results

### 2.1. Evaluation of Biofilm Thickness on Flat Sheet Membrane Using Microscopy

#### 2.1.1. QQ’s Effects on PTFE Membrane

**Figure 1:**
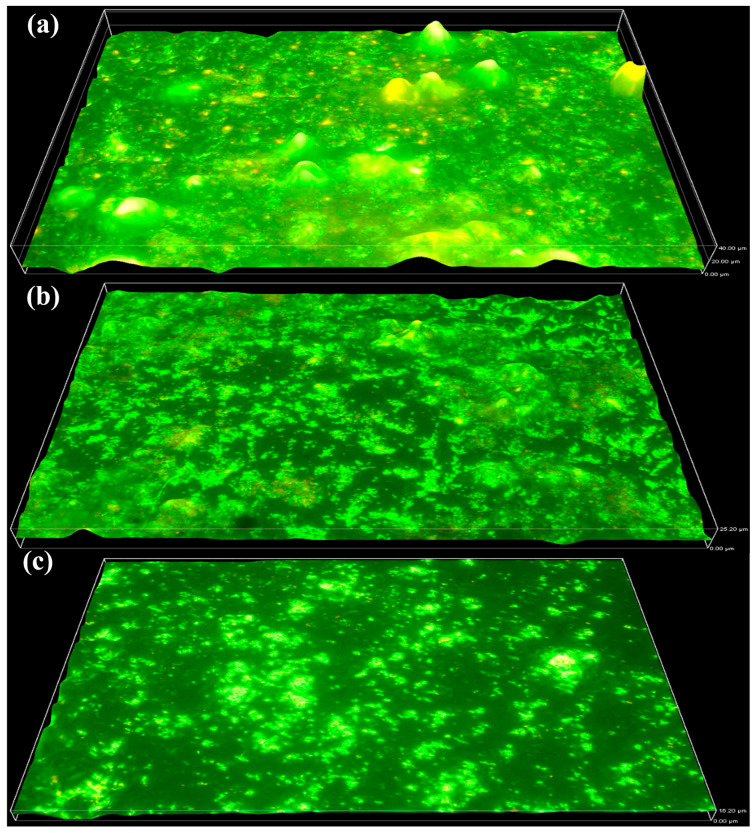
Biofilm thickness on the PTFE membrane is increasing: (MBR-1) from 0 µm to 40 µm by week 13 (**a**), (MBR-2) 25.2 µm by week 13 (**b**), and (MBR-3) to 16.2 µm by week 13 (**c**).

The biofilm formation on the PTFE membrane was examined using fluorescence microscopy. Figure 1a represents the results of MBR-1, which was operated as a control reactor. MBR-1 showed a pronounced increase in biofilm growth over time, reaching a thickness of 40 µm by the end of week 13. The significant accumulation, growth and biofilm formation indicate that the operating conditions, including the membrane surface, were highly favourable for microorganisms. MBR-2 was operated with 2 g L^−1^ QQ bacteria. As shown in Figure 1b, the maximum biofilm thickness observed at week 13 was 25.2 µm, which was markedly lower than that of the control operation. These results indicate that the QQ interface effectively disrupted the QS pathway, thereby leading to a substantial reduction in biofilm formation. MBR-3 exhibited the least biofilm formation and was operated with 5 g L^−1^ QQ bacteria. By the end of week 13, the maximum biofilm thickness reached 16.2 µm, which was substantially lower than that observed in both MBR-1 and MBR-2. This pronounced reduction demonstrates that the higher dosage of QQ bacteria effectively disrupted QS and mitigated biofilm development.

Table 1 shows the statistical analysis of the PTFE membrane based on five distinct sampling points. MBR-1 exhibited the highest recorded value (37.96 ± 2.4). MBR-2, which was subjected to QQ bacteria, showed a reduced value (25 ± 1.3), while MBR-3 demonstrated the greatest reduction (15.1 ± 0.99). These results indicate a progressive decrease due to the intervention of QQ, and the statistical findings also support the corresponding 3D surface images.

**Figure 2:**
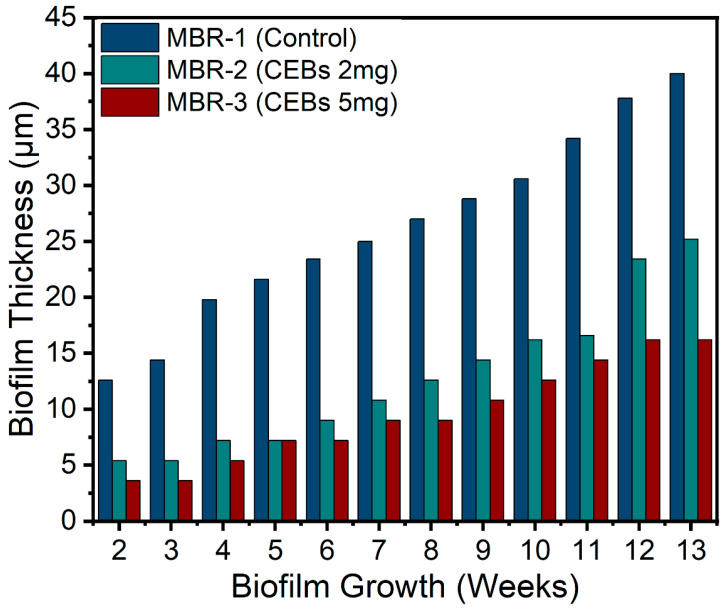
Biofilm thickness on the PTFE membrane shows a progressive increase over time.

Biofilm formation on the surface of the PTFE membrane was observed throughout the operation on a weekly basis. Figure 2 illustrates the progressive growth of biofilm thickness under three different operation conditions, indicating continuous biofilm development. However, the control operation exhibited substantially higher biofilm accumulation, with the thickness increasing from approximately 12.6 µm in week 2 to about 40 µm by week 13. In contrast, reactors supplemented with 2 g L^−1^ cell-entrapping beads (CEBs) showed substantially reduced biofilm growth, demonstrating a moderate inhibitory effect. When MBR-2 was compared with MBR-1, an overall average reduction of 51.3% in biofilm thickness was observed, attributed to QQ intervention. MBR-3 exhibited even greater biofilm reduction compared to both other operations, indicating a dose-dependent inhibitory effect of QQ bacteria on biofilm formation, achieving a 63.5% reduction compared to the control.

#### 2.1.2. Influence of QQ on PS Membrane

**Figure 3:**
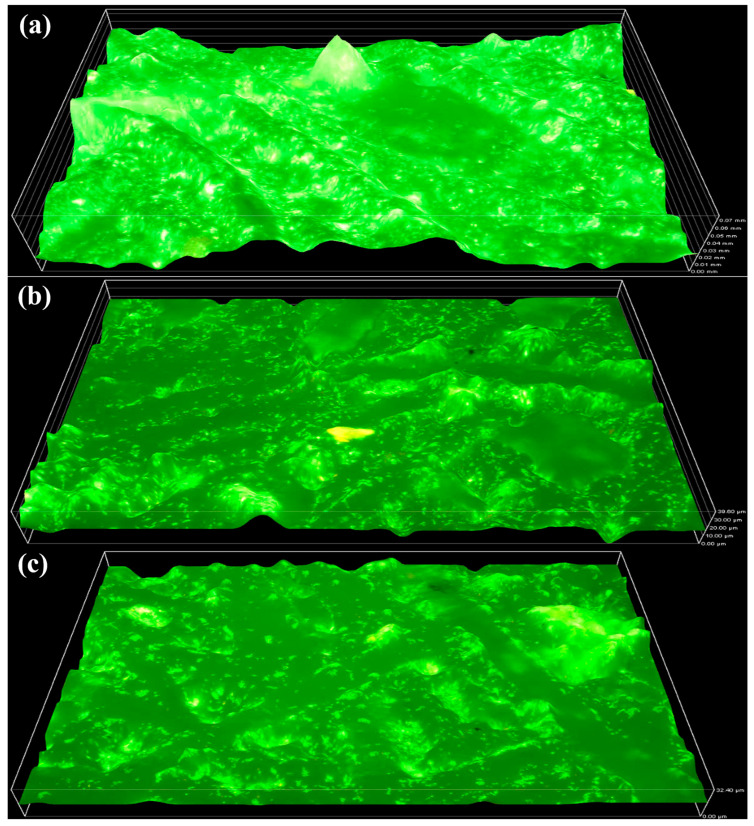
Biofilm thickness on the PS membrane is increasing: (MBR-1) from 0 µm to 70 µm by week 13 (**a**), (MBR-2) 39.6 µm by week 13 (**b**), and (MBR-3) to 32.4 µm by week 13 (**c**).

Biofilm formation on the surface of the PS membrane was observed to be more pronounced than on the PTFE membrane. This difference can be primarily attributed to the membrane material and its higher surface roughness, which provides a more favourable surface for microbial adhesion, growth, and subsequent biofilm maturation. Figure 3a illustrates the control operation, in which the maximum biofilm thickness reached 70 µm by the end of week 13. The biofilm appears dense and well-established, as evidenced by the continuous surface coverage and compact microbial accumulation observed in the microscopic images, indicating biofilm maturation and strong microbial attachment to the membrane. In contrast, Figure 3b shows reduced biofilm formation over the same time frame, with a measured thickness of 39.6 µm, corresponding to MBR-2. This reduction suggests that QQ activity inhibited microbial communication, resulting in slower biofilm accumulation and a less compact biofilm structure. Similarly, MBR-3 (Figure 3c) exhibits the least biofilm development among the three operations. The relatively low biofilm thickness of 32.4 µm indicates weaker microbial adhesion and delayed biofilm maturation on the membrane surface.

Table 1 illustrates the statistical analysis of the PS membrane. The control reactor exhibited the highest biofilm thickness among all tested samples. MBR-2 showed a noticeable reduction in biofilm thickness compared with the control, whereas MBR-3 demonstrated the lowest recorded value, indicating a greater reduction attributed to the increased dosage of QQ bacteria.

**Figure 4:**
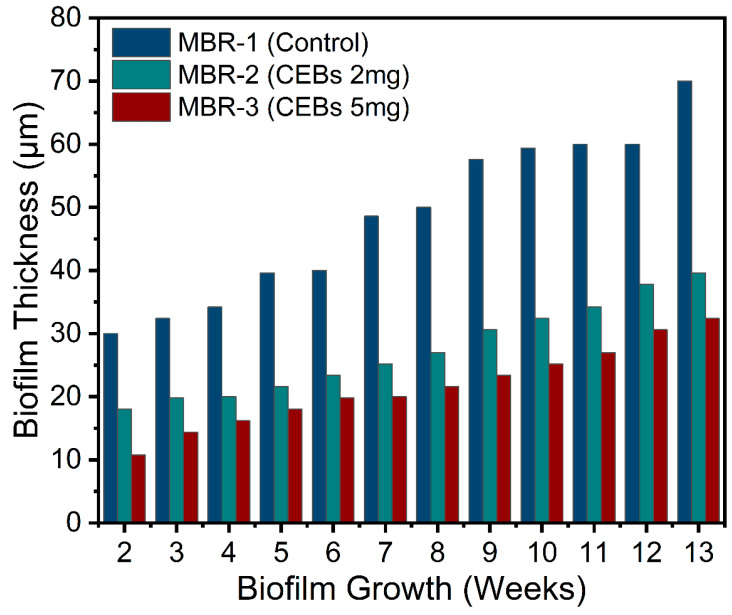
Biofilm thickness on the PS membrane shows a progressive increase over time.

The overall biofilm thickness increased over time in all operations, indicating a progressive development of biofilm. However, the ratio of biofilm formation was quite different, and this difference was completely dependent on operational conditions. Figure 4 reveals that the biofilm thickness showed a clear and consistent difference among all three MBRs. MBR-1 exhibited the highest biofilm thickness throughout the operation. Biofilm growth increased steadily from 30 µm at week 2 to 70 µm by week 13, demonstrating rapid biofilm accumulation and continuous maturation. In contrast, MBR-2 showed a noticeably reduced biofilm growth compared to the control. Biofilm thickness increased gradually from around 18 µm at week 2 to approximately 39.6 µm by week 13. Although biofilm formation was not completely suppressed, it consistently had a reduced thickness and achieved an average reduction of 43.4% compared to MBR-1, indicating partial inhibition of biofilm development. This suggests that the addition of QQ bacteria successfully interfered with microbial communication mechanisms. MBR-3 demonstrated the strongest QQ-inhibitory effect on biofilm formation throughout the operational period, and biofilm thickness increased from 10.8 µm at week 2 to only 32.4 µm by week 13, achieving a maximum reduction of 55.4%. The slower growth rate and reduced final thickness indicate that the higher CEB dosage disrupted QS activity more effectively, resulting in weaker microbial adhesion, reduced EPS production, and delayed biofilm maturation.

#### 2.1.3. Comparison of Filtration Resistances

**Figure 5:**
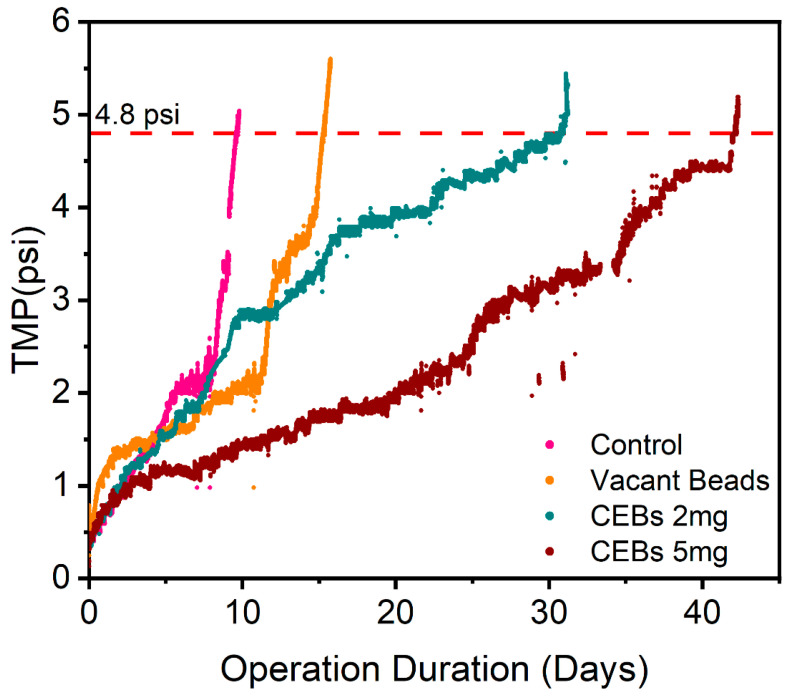
TMP profile across the different operations.

To prove the above-mentioned results, the MBRs were operated with and without QQ bacteria. A new membrane module was immersed in each MBR and operated until the membranes got fouled and TMP reached a maximum threshold (4.8 psi = 33 kPa) under steady-state treatment conditions. Figure 5 illustrates the TMP profiles of four different operations, all of which were operated under different conditions. The first operation was a control operation without PVA-SA beads and QQ intervention; in this system, the membrane got fouled in 9.6 days. The second operation was performed with vacant beads that physically interact with the membrane because of aeration. Due to this physical interaction, bacteria take more time to accumulate on the membrane surface. As a result, this operation takes a longer duration of 15.3 days, which represents an improvement of 58.9% compared to the control operation. The third operation was performed with 2 g L^−1^ CEBs, which shows the QQ intervene, thereby demonstrating that membrane performance was improved and that the TMP was delayed and reached the threshold in 31.5 days, showing a 228.6% improvement. Similarly, in the fourth operation, where 5 g L^−1^ CEBs were used, it shows even better performance in terms of operation duration, indicating a further enhancement of 42 days, showing an overall 338.2% improvement.

### 2.2. EPS Content on Membrane Surface and Sludge

**Figure 6:**
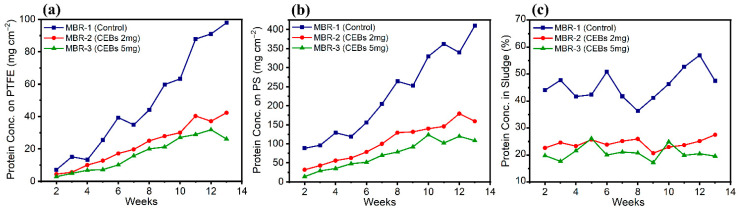
Concentration of protein (**a**) on PTFE; (**b**) on PS; (**c**) in the sludge of the bioreactor.

The concentration of EPS protein was determined on the surface of membranes and within the sludge. Figure 6a revealed the protein concentration on the surface of the PTFE membrane across the three different operational conditions. MBR-1 showed a rapid and continuous increase over time, reaching a peak by the end of week 13 at 97.8 mg cm^−2^, which was substantially higher than other operations. In contrast, MBR-2 exhibited the lowest protein concentration throughout the operation, reaching a maximum of 42.3 mg cm^−2^ due to the intervention of QQ. This shows a significant reduction of 53% compared to the control. In MBR-3, the protein concentration was further suppressed owing to the increased dose, resulting in a 65% reduction in protein content. Figure 6b shows the protein concentration on the surface of the PS membrane, which provides a more favourable surface for bacterial accumulation and growth compared to the PTFE membrane. Consequently, more protein was found on the PS membrane. MBR-1 exhibited the maximum protein concentration of 400.9 mg cm^−2^ by week 13, which was approximately 3-fold greater than that observed for the PTFE membrane. In contrast, the MBR-2, which contained QQ bacteria, showed a substantially lower protein production on the membrane surface, reaching 159.2 mg cm^−2^ in week 13. This corresponded to an overall reduction of 54.4%, which was lower than that of the control. Similarly, MBR-3, which contained a higher dose of QQ bacteria, showed further suppression of protein, with a maximum protein concentration of 108.3 mg cm^−2^ in week 13. Consequently, this system achieved an even greater reduction of 68.3%. Figure 6c shows the protein concentration in the overall sludge of the bioreactor, which was directly measured from each individual reactor and is independent of membrane material. For each reactor, PTFE and PS membrane samples were collected separately, while the sludge sample represents the bulk mixed liquor of the corresponding reactor. The weekly trends across all operational conditions exhibited noticeable fluctuations, most likely due to the continuous suspension and mixing of sludge within the reactor. Despite these fluctuations, an overall comparison between the control and the QQ operations revealed significant differences under all three conditions. MBR-2 and MBR-3 achieved protein reductions of 47% and 54.7%, respectively. Although the difference between the two QQ reactors was relatively small, this can be logically attributed to the suspended nature of the sludge, which leads to homogenization and limits protein accumulation, thereby reducing the distinguishable impact of higher QQ bacterial dosage.

**Figure 7:**
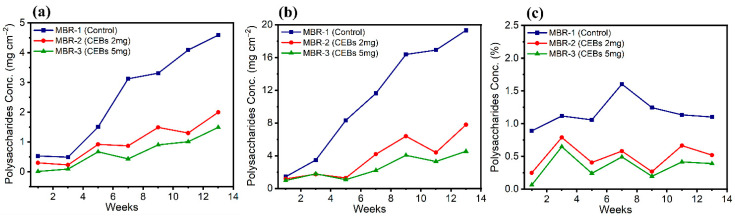
Concentration of polysaccharides (**a**) on PTFE; (**b**) on PS; (**c**) in sludge.

The EPS polysaccharide concentration was quantified on both membrane surfaces and within the sludge. Figure 7a shows the distribution of polysaccharide content on the PTFE membrane, highlighting significant differences among the three operating conditions. The polysaccharide content was substantially higher in the control reactor. A notable reduction in EPS polysaccharides due to QQ interference was observed, with reductions of 59.6% in MBR-2 and 73.8% in MBR-3. Similarly, the same trend was observed on the PS membrane in Figure 7b. However, it exhibited a higher polysaccharide content, as its material is more favourable for bacterial accumulation. Despite this tendency, QQ demonstrated a substantial effect in reducing EPS polysaccharide synthesis. In this regard, MBR-2 attained a 65.2% reduction, whereas MBR-3 achieved a 76.9% reduction. Figure 7c illustrates the concentration of EPS polysaccharides in the sludge, indicating a substantial decrease across the modified MBR systems. MBR-2 exhibited a 53.3% reduction, while MBR-3 demonstrated a more pronounced reduction of 69.9%, suggesting enhanced control of EPS polysaccharides.

### 2.3. Sludge Properties

**Figure 8:**
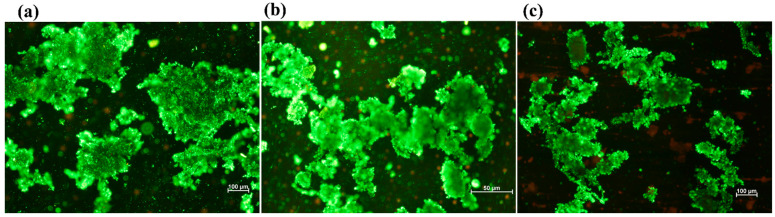
Microscopic images of sludge flocs in (**a**) MBR-1, (**b**) MBR-2, and (**c**) MBR-3.

The above-mentioned results demonstrate that QQ affected EPS production, biofilm formation, and membrane filtration resistance. In particular, the quantified reduction in EPS suggests a direct alteration in QS-regulated microbial metabolic activities. Since EPS plays a critical role in maintaining the structural integrity and cohesion of sludge flocs, its reduction likely weakened the floc matrix. This structural weakening is supported by the observed decrease in membrane filtration resistance and the microscopic evidence of smaller and less compact sludge aggregates. Therefore, the combined evidence from EPS analysis, filtration performance, and morphological observations indicates that QQ interfered with QS-regulated microbial metabolism, suppressed EPS secretion, and consequently led to reduced sludge floc size. Figure 8a shows that the sludge flocs are large, dense and compact with strong aggregation, which is characteristic of well-developed and stable sludge flocs. Meanwhile, Figure 8b shows that the flocs appear less compact, smaller in size and fragmented because of QQ activity, suggesting partial disruption of microbial aggregation. Similarly, in Figure 8c, in which a higher QQ dose was used, there is a clear reduction in overall floc size and cohesion and a more dispersed distribution of fine and loosely bound microflocs. Table 2 shows that the statistical analysis of the floc size presents an approximate range of equivalent diameter (µm) and estimated area (µm^2^) under different conditions. Each entry reports a consistent sample count alongside the mean and standard deviation for both physical properties, ensuring statistical reliability and comparability among the datasets and indicating a progressive decrease in both dimensions from MBR-1 to MBR-3.

### 2.4. Comparison of Reactor Performance

**Figure 9:**
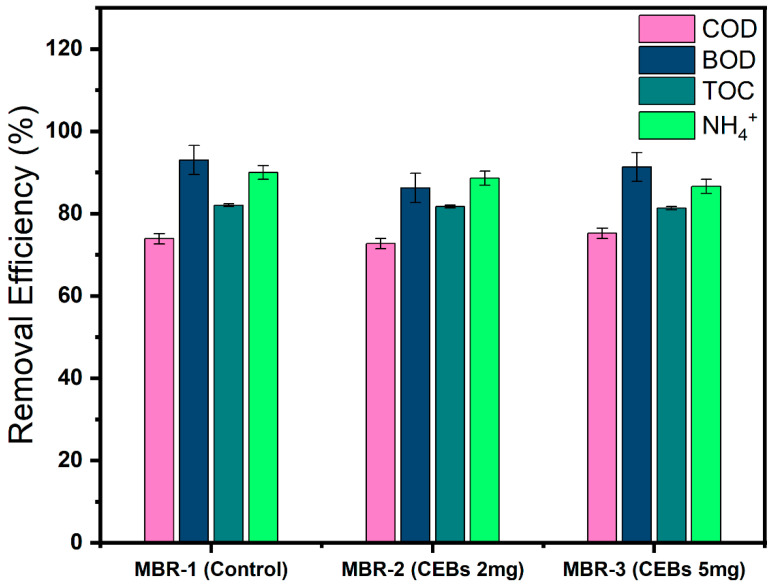
Comparison of pollutant removal.

The reactor’s performance was evaluated based on pollutant removal. Although this study was primarily focused on biofilm formation and EPS reduction, the ultimate objective of sewage treatment remains effective pollutant removal. Since the reactors were operated under different conditions, it is important to take reactor performance into account in terms of pollutant removal to ensure a comprehensive evaluation of system efficiency. Figure 9 illustrates that the COD removal efficiency across all reactors ranged from 73 to 75%, with no significant difference throughout the operational period. The BOD removal efficiency was consistently higher, reaching 93% in MBR-1, while MBR-2 and MBR-3 achieved 86% and 91%, respectively. Similarly, the TOC removal remained stable, with an efficiency of 80% in all reactors. The ammonia removal efficiency in MBR-1 was slightly higher, at approximately 90%, whereas the QQ reactors achieved about 87%, suggesting marginally enhanced nitrification in MBR-1. Tables S1–S4 present detailed information on the influent concentrations of COD, BOD, TOC, and ammonia, as well as their removal efficiencies across the different operations.

## 3. Discussion

The development of biofilm thickness was highly consistent on the surface of the PTFE membrane, and it continued to increase over time in all operations. However, this increase was more pronounced in the control operation because of the lack of an antifouling strategy. Previous studies indicate that the PTFE membrane provides a favourable surface for microorganisms to adhere to due to its hydrophobic nature and surface roughness, which promote initial adhesion and subsequent biofilm maturation [32,33]. It was also noticed that the absence of a biofilm control approach enables stopping QS; as a result, bacteria produce more EPS, which leads to the formation of denser and more structurally stable biofilm [34]. In MBR-2, there was a notable decrease in biofilm formation compared to the control, which is strong evidence that the presence of QQ bacteria successfully intervenes with the QS pathway and also minimizes EPS secretion, resulting in less compact biofilms on the membrane surface [35]. QQ primarily works during the early stages of biofilm development, particularly during initial bacterial attachment and biofilm formation. QS-regulated gene expression is strongly involved in EPS production and structural organization during biofilm maturation. Therefore, degradation of AHL signalling molecules by QQ bacteria disrupts intercellular communication before the establishment of a stable biofilm matrix. This early interference limits the formation of EPS, which ultimately results in thinner and structurally weaker biofilms [36]. The greatest fouling reduction was observed in MBR-3, which clearly demonstrates the dose-dependent effect of QQ bacteria. At a higher concentration of QQ bacteria, a greater reduction in biofilm thickness was achieved throughout the operation. It also enhanced the degradation rate of QS signalling molecules, leading to stronger suppression of biofilm adhesion and maturation. Similar dose-dependent relationships between QQ activity and biofilm control have been reported in MBR, where increased QQ bacterial activity improved mitigation of membrane biofouling [7,37]. These results suggest that a sufficient QQ dose can effectively maintain QS disruption over long-term operation. The results obtained from the PS membrane under control operation clearly demonstrate that the accumulation of bacteria on the PS membrane surface was higher than that observed on the PTFE membrane. Both membranes have distinct physicochemical properties, which provide a useful contrast for evaluating material-dependent biofilm behaviour and QQ performance. This behaviour can be attributed to the relatively lower hydrophobicity of PS compared to PTFE, which enhances microbial adhesion due to differences in surface energy and membrane-microbe interactions. In contrast, PTFE, being more hydrophobic, limits initial bacterial attachment under the same operating conditions. This contrast allows for the assessment of how surface variations influence bacterial adhesion, EPS deposition, and subsequently biofilm structure. By comparing these two materials under identical operational conditions, the study elucidates how membrane-specific properties control QS activity and improve biofilm compactness and membrane filtration performance. However, regarding QQ performance, bacterial accumulation was strongly influenced by operational conditions, particularly the presence and dosage of QQ bacteria, which affected biofilm formation and EPS production within the reactor system. In MBR-1, the biofilm thickness continuously increased, which indicates accumulation and continuous maturation. This behaviour typically corresponds to conventional MBR, where biofilm growth is governed by microbial adhesion, and EPS secretion and QS interaction occur among microbial communities. These processes ultimately promote microbial attachment, growth, and proliferation, leading to the formation of a dense cake layer on the membrane surface. This uncontrolled biofilm development and rapid fouling align with previous studies [38,39]. In contrast, MBR-2, where QQ bacteria were introduced, resulted in a noticeable reduction in biofilm thickness. Similar decreases in biofilm thickness have been reported in previous research using QQ bacteria, where degradation of AHL molecules disrupted bacterial communication and subsequently limited EPS production and biofilm maturation [40]. The maximum QQ dosage in MBR-3 exhibited the most pronounced inhibitory effect. The slower growth rate and the lower final biofilm thickness show that a higher concentration of QQ cells more effectively disrupted QS pathways, resulting in weaker initial microbial adhesion and a significant decrease in EPS production. These findings support the application of the QQ technique as a promising and environmentally benign approach for long-term biofouling mitigation in MBR. These results are consistent with previously reported studies demonstrating that an optimized dose of QQ bacteria effectively suppresses biofilm formation [41].

The TMP is a very important factor in measuring the membrane filterability in MBR, because it is directly linked to membrane fouling. The variation in TMP was clearly observed in Figure 5 for the different operations. In the control MBR, the initial rise in TMP is most likely due to passive adsorption of organics, which allows microorganisms to grow on the membrane surface and block the pores externally, while after a certain time, an abrupt rise in TMP indicates that the pores are blocked internally as well [42]. The enhancement of the operation with vacant beads is attributed to physical scouring effects induced by the bead’s membrane interaction. The hydrodynamic conditions were controlled by maintaining a constant aeration rate of 3 L min^−1^ and a fixed bead loading. At this aeration rate, effective bead fluidization and continuous contact with the membrane surface were observed, indicating active mechanical interaction between the beads and the membrane surface. Such physical interaction has been widely reported to inhibit initial bacterial attachment on membrane surfaces, thereby delaying the biofouling. Previous studies have demonstrated that effective fouling mitigation can be attained through the incorporation of suspended plastic carriers, sponge carriers or granular material, which reduced the cake layer and delayed biofilm formation [4,31,43]. The significant improvement in the third operation indicates the effectiveness of QQ bacteria as a biological fouling control technique. This approach disrupts the QS signal pathway, particularly AHL molecules, which are responsible for EPS production, microbial accumulation and ultimately biofouling. It is possible to delay TMP and enhance membrane performance to provide strong evidence of the success of QQ bacteria in becoming a barrier to bacterial communication [44,45]. Further improvement suggests a dose-dependent QQ effect, where an increased dose of QQ bacteria releases more enzymes, thereby enhancing the degradation of signal molecules and leading to the suppression of biofouling. Previous studies align with these findings and indicate that, beyond the air scouring effect, optimizing the QQ dose plays a significant role in mitigating membrane biofouling [46].

The results draw a clear mechanistic connection between membrane material properties, biofilm characteristics, EPS production, and filtration performance. Membranes with a stronger hydrophobic nature promote more rapid bacterial adhesion and then start EPS synthesis, which contributes to structural compactness and increased biofilm thickness. This compact biofilm increases hydraulic resistance, resulting in a more rapid rise in TMP. On the other hand, QQ activity disrupts QS pathways, which reduces EPS synthesis and results in a less compact biofilm structure, ultimately improving membrane filterability and slowing TMP progression [47]. Therefore, membrane material properties and QQ activity collectively regulate fouling behaviour through their combined influence on biofilm structure and composition.

The secretion of EPS protein and polysaccharides is primarily due to microbial metabolic activity. In MBR-1, the continuous increase in EPS content on both membranes (PTFE and PS) indicates active synthesis of protein and polysaccharides, which ultimately leads to biofilm maturation and stabilization [38]. In contrast, MBR-2 and MBR-3 demonstrate that QQ directly mitigates EPS biosynthesis by disrupting the QS pathway and regulating the extracellular enzyme activity [48]. The higher EPS concentrations found on the PS compared to the PTFE are attributed to differences in surface interactions rather than biofilm thickness alone. The surface roughness and hydrophobicity of PS promote mechanical entrapment of colloidal matter with a rough surface, enhance contact and facilitate stronger adsorption of EPS through hydrophobic interactions [14]. In the sludge, the EPS content varied, because the sludge was suspended in the reactor, and dilution also had an effect. However, as far as the QQ effect was concerned, a pronounced reduction in EPS was observed in the sludge. MBR-3 exhibited a stronger reduction in EPS on both the membranes and in the sludge, which can be attributed to its higher abundance of QQ bacteria. Consequently, EPS synthesis was inhibited in a dose-dependent manner due to QQ activity. These findings align with previously reported studies, indicating that EPS production plays a crucial role in biofilm stabilization, whereas its impact on suspended sludge flocs is comparatively less significant [14,49].

The results of this study clearly demonstrate that QQ has a strong impact on sludge characteristics by reducing EPS content, biofilm formation and membrane filtration resistance. This observation indicates that QQ influences microbial metabolic activity, which ultimately leads to a decrease in sludge floc size [50]. This strongly supports the effect of QQ, which enhanced the floc fragmentation and reduced sludge production, likely due to interference in the QS pathway. These results also align with previous studies, confirming that QQ leads to weakening of the sludge floc structure, which ultimately reduces biofilm formation [51,52]. Despite these structural changes, QQ did not have any adverse effect on pollutant removal in the MBRs. The system maintained comparable treatment efficiency to previously reported studies [53], indicating that QQ application can mitigate membrane fouling without compromising reactor performance.

## 4. Materials and Methods

### 4.1. Chemicals

PVA 2270° (UNI-Chem, Lahore, Pakistan) with a purity of 99% was used. Sodium alginate was purchased from Fisher (Loughborough, UK). Boric acid was purchased from AppliChem (Darmstadt, Germany), and anhydrous calcium chloride (CaCl_2_) and sodium sulphate (Na_2_SO_4_) were purchased from Merck (Darmstadt, Germany). R2A broth medium was purchased from Neogen (Heywood, UK). The fluorescent dyes SYTO 9 and propidium iodide were both purchased from Invitrogen (Eugene, OR, USA).

### 4.2. Lab-Scale MBR Design, Setup, and Operation

**Figure 10:**
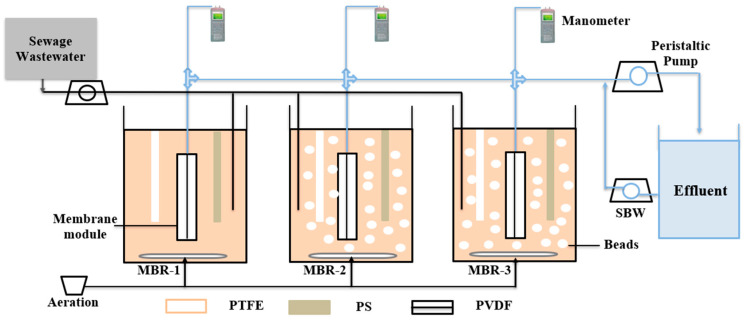
Schematic diagram of the MBR.

Three MBRs were operated in parallel, with a working volume of 4 L, a hydraulic retention time (HRT) of 5.1 h and a target MLSS concentration of 8 g L^−1^. MBR-1 served as a control reactor without beads, whereas MBR-2 and MBR-3 were operated with QQ bacterial concentrations of 2 g L^−1^ and 5 g L^−1^, respectively. The filling ratio of the beads was set at 1% (*v*/*v*) of the total reactor volume. All MBRs were fed with municipal wastewater obtained from a local municipal treatment plant with an organic loading rate (OLR) ranging from 1.7 to 2 kg COD m^−3^*day^−1^ and a food-to-mass ratio (F/M) of 0.2 kg COD kg MLSS^−1*^ day^−1^. Aeration was provided at the bottom of each reactor to maintain an average dissolved oxygen (DO) concentration of 2.9 mg L^−1^. Each MBR was equipped with hollow-fibre PVDF ultrafiltration membranes (pore size 0.1 μm, PHILOS, Gwangmyeong-si, Korea). The membranes were connected to a peristaltic pump (ISM833C, Odessa, TX, USA) to withdraw the treated wastewater as permeate. The filtration process was conducted over 10 min, followed by a 1 min standard backwash (SBW). The TMP was measured by a SPER scientific data logging manometer (840099, limited liability company, San Bernardino, CA, USA), which took one reading every two minutes. Flat sheet membranes, including microfiltration membranes PTFE with a pore size of 0.2 µm (Sartorius Göttingen, Germany) and PS (Alfa Laval Mid Europe GmbH, Hamburg, Germany), were additionally submerged in each MBR. These membranes were not operated for filtration. A schematic representation of the operational setup is shown in Figure 10. The scientific purpose of immersing PTFE and PS membranes in the MBR without filtration was to assess intrinsic microbial-material interactions, biofilm formation dynamics, and the adsorptive attachment behaviour of EPS on different membrane materials under identical biological conditions. In a conventional filtration module, it is difficult to periodically collect small membrane samples for analysis without damaging the entire module. Therefore, the membranes were intentionally immersed in the reactor to enable systematic and continuous sampling and direct observation of biofilm development over time without disrupting reactor operation.

### 4.3. Bead Preparation and Immobilization of QQ Bacteria

PVA and SA were dissolved in deionized water at concentrations of 0.08 g mL^−1^ and 0.01 g mL^−1^, respectively. The mixture was heated in a drying oven at 105 °C for 4 h to obtain a clear and homogeneous polymer solution. The resulting solution was then dripped into a first cross-linking solution containing boric acid (0.07 g mL^−1^) and calcium chloride (0.04 g mL^−1^), with the temperature being maintained between 35 and 40 °C. The prepared beads were soaked in the same solution for 30 min and then washed with deionized water. The beads were then transferred to a second cross-linking solution containing sodium sulfate (0.07 g mL^−1^) and kept for 2 h. The PVA-SA beads were spherical in shape, with an average diameter of 4 mm. The mechanical stability of the beads was evaluated using a centrifuge (UNIVERSAL 320R, Hettich, Tuttlingen, Germany).

*Rhodococcus* sp. BH4 (Accession no. CP014941) was used as the QQ bacterium, which was obtained from Forman Christian College (A Chartered University), Lahore, Pakistan. The bacterial strain was initially cultivated on agar plates, from which a fresh colony was transferred into 50 mL of sterilized R2A broth as the primary culture. The culture was incubated on an orbital shaker (Certomat MO II, Sartorius, Göttingen, Germany) at room temperature for 1 day. Subsequently, a secondary culture was prepared by adding the primary culture to 450 mL of R2A broth, which was again incubated on the shaker for 1 day. After incubation, the bacterial suspension was harvested by centrifugation at 5000 rpm for 10 min at room temperature. The resulting bacterial pellet was resuspended in 2 mL of deionized water and incorporated into the PVA-SA solution. The concentrations of 2 g L^−1^ and 5 g L^−1^ refer to the loading of QQ bacterial biomass entrapped within the beads for MBR-2 and MBR-3, respectively. The mixture was thoroughly homogenized to ensure uniform bacterial distribution. The prepared solution was then used for the formation of cell-entrapping beads (CEBs) with lower (MBR-2) and higher amounts (MBR-3) of QQ bacteria. The bacterial concentration was determined by the dry weight method after filtration and drying at 60 °C for 30 min.

### 4.4. Assessment of Biofilm Formation and Sludge Properties Using Fluorescence Microscopy

Fluorescence microscopy (ECLIPSE LV100, Nikon, Chiyoda-ku, Japan) was used to evaluate the biofilm thickness by observing the accumulation of the microbial community on the surface of PTFE and PS membranes. Flat sheet membrane samples were collected on a weekly basis to monitor the biofilm development. For this purpose, small pieces of membrane were excised, rinsed with tap water, placed on a glass slide, and stained using the LIVE/DEAD assay (BacLight TM Bacterial Viability Kits, Invitrogen, Karlsruhe, Germany), where SYTO 9 stains live bacteria in green and propidium iodide stains dead bacteria in red. The prepared samples were then examined using three-dimensional (3D) imaging to determine biofilm thickness at 40× magnification, providing a detailed qualitative assessment of membrane fouling. Sludge flocs were observed weekly under fluorescence microscopy using the LIVE/DEAD assay to evaluate cell viability and structural integrity within the floc matrix.

### 4.5. Statistical Analysis

Descriptive statistics were used to analyse the obtained 3D images to assess biofilm thickness. For this purpose, five measurement points (*n* = 5) were selected from each membrane sample. These points represent technical measurement replicates obtained from the same sample. The mean and standard deviation (mean ± SD) were calculated to describe the spatial variability within each sample. Similarly, for the sludge characterization, the sludge floc equivalent diameter (µm) and the estimated area (µm^2^) were analysed. For this analysis, five independent measurements per sample were recorded, and the mean ± standard deviation was calculated accordingly.

### 4.6. Extraction and Measurement of EPS

For protein extraction, 1.5 cm long × 1.0 cm wide membrane samples of both PTFE and PS and 2 mL of sludge were collected weekly from the reactors. The membrane pieces and sludge sample were rinsed with tap water and potassium phosphate-buffered solution (pH 7) to remove loosely attached substances. Then, the samples were incubated in 1 M NaOH for 15 min at room temperature, followed by lysing the samples at 100 °C for 5 min. Subsequently, they were cooled on ice and centrifuged again (FRESCO-17, Osterode, Germany) at 7000 rpm for 30 min [54]. The concentration of EPS protein was determined using the Lowry method [55], with absorbance being measured at 570 nm using a Beckman DU 600 spectrophotometer (Fullerton, CA, USA).

For EPS polysaccharide extraction, membrane samples of the same dimensions and sludge were used. The samples were placed in 4 mL of 1 mM Na-EDTA for 4 h, followed by sonication using a JX-031S ultrasonic cleaner (Skymen Private Coorporation Ltd., Shenzhen, China) for 30 min, using a water bath to facilitate the detachment of polysaccharide content from the samples. Then, the samples were incubated for an additional 4 h. After incubation, 2 mL of the sample was collected and centrifuged at 13,000 rpm for 30 min. The collected supernatant was used for further analysis. The polysaccharide concentration was determined using the photometric phenol-sulfuric acid method [42]. The polysaccharide content was measured at 490 nm.

### 4.7. Analytical Method

The total organic carbon (TOC) was analysed using the TOC analyser DIMATOC-100 (Diamtech, Essen, Germany) according to DIN EN 12260 [56]. NH_4_^+^ was measured with a Shimadzu UV-2450 spectrophotometer (Tokyo, Japan) according to the European standard procedure DIN 38406 E5 and EN ISO 6878:2004, respectively [57,58]. The chemical oxygen demand (COD) was measured according to DIN ISO 15705:2003-01 (Merk cell test) [59]. The biological oxygen demand (BOD) was measured using Respirometer BSBdigi from Selutec (Hechingen, Germany), according to EN ISO 9408 [60]. The MLSS was determined according to the standard method [61].

## 5. Conclusions

This study demonstrates the effectiveness of QQ bacteria *Rhodococcus* sp. BH4 in the mitigation of membrane biofouling across different membrane materials. Its impact on biofilm thickness, EPS production, sludge properties and overall treatment performance was investigated, leading to the following conclusions:

- The biofilm thickness on the PTFE and PS membranes was reduced by 63.5% and 55.4%, respectively.
- PVDF membrane filtration cycle was increased by 338.2% with QQ entrapment in PVA beads.
- Furthermore, the results demonstrate that the secretion of EPS protein and polysaccharides was substantially reduced on both the membrane surface and in the sludge.
- QQ intervention reduced the sludge floc size, leading to improved sludge settleability while maintaining stable effluent quality.

The findings of this study indicate that the optimized dose of QQ bacteria achieved the maximum reduction, highlighting its potential applications in MBR and its role in optimizing biofilm thickness and controlling biofouling.

## Figures and Tables

**Table 1:** Statistical analysis of biofilm thickness (µm) on PTFE and PS membranes.

Reactor	PTFE Week 13 (µm)	PS Week 13 (µm)
MBR-1 (Control)	37.96 ± 2.4	63.9 ± 5.6
MBR-2 (CEBs 2 mg)	25 ± 1.3	39.3 ± 0.9
MBR-3 (CEBs 5 mg)	15.1 ± 0.99	32 ± 1.5

Values represent mean ± standard deviation (*n* = 5 thickness points per sample).

**Table 2:** Statistical analysis of sludge flocs.

Property	MBR-1 No. of Count|Mean ± SD	MBR-2 No. of Count|Mean ± SD	MBR-3 No. of Count|Mean ± SD
Equivalent Diameter (µm)	5/(3.85 ± 1.12) × 10^2^	5/(1.91 ± 0.15) × 10^2^	5/(7.29 ± 0.87) × 10^1^
Estimated Area (µm^2^)	5/(1.05 ± 0.62) × 10^5^	5/(2.88 ± 0.43) × 10^4^	5/(4.23 ± 0.98) × 10^3^

Values represent mean ± standard deviation (*n* = 5 points of sample).

## Data Availability

Data are contained within the article and Supplementary Materials.

## Supplementary Materials

The following supporting information can be downloaded at https://www.mdpi.com/article/10.3390/molecules31061022/s1: Table S1: Removal efficiency of COD across three different operations; Table S2: Removal efficiency of BOD across three different operations; Table S3: Removal efficiency of TOC across three different operations; Table S4: Removal efficiency of ammonia across three different operations.
