# Supplementary material for: Mechanistic Insights into Quorum Quenching-Mediated Control of EPS and Biofilm Formation in Submerged MBR
Source: Molecules. 2026 Mar 19;31(6):1022. doi: 10.3390/molecules31061022 (PMC13029297; doi:10.3390/molecules31061022)
Supplement: Supplementary file 1 [file molecules-31-01022-s001.zip › molecules-4184500-supplementary.pdf]

Supplementary Notes (supporting information to the full text paper)

Mechanistic insights into quorum quenching-mediated control of EPS and biofilm formation in submerged MBR

Noman Sohail [1,*] and Marion Martienssen [1]

[1]Chair of Biotechnology of Water Treatment, Brandenburg University of Technology Cottbus/Senftenberg, 03046 Cottbus, Germany

Removal efficiency

Table S1: Removal Efficiency of COD across three different operations

Days of operation	Influent mg L^{-1}	Effluent (MBR-1) mg L^{-1}	Removal efficiency MBR-1 (%)	Effluent (MBR-2) mg L^{-1}	Removal efficiency MBR-2 (%)	Effluent (MBR-3) mg L^{-1}	Removal efficiency MBR-3 (%)
1	355	153	56.9	163	54.1	103	71
14	360	78	78.3	99	72.5	96	73.3
29	355	109	69.3	86	75.8	114	67.9
42	374	130	65.2	125	66.6	128	65.8
60	436	38	91.3	92	78.9	68	84.4
81	396	73	81.6	89	77.5	68	82.8
93	365	65	82.2	59	83.8	44	87.9
103	430	144	66.5	119	72.2	135	68.6
Average	384	98.75	73.9	104	72.7	94.5	75.2

Table S2: Removal Efficiency of BOD across three different operations

Days of operation	Influent mg L^{-1}	Effluent (MBR-1) mg L^{-1}	Removal efficiency MBR-1 (%)	Effluent (MBR-2) mg L^{-1}	Removal efficiency MBR-2 (%)	Effluent (MBR-3) mg L^{-1}	Removal efficiency MBR-3 (%)
9	170	7.9	95.4	15	91.2	9.1	94.6
42	114	11	90.4	17	85.1	9	92.1
60	167	9.1	94.6	27	83.8	13	92.2
93	110	9	91.8	17	84.5	15	86.4
Average	140	9.3	93	19	86.2	11.5	91.3

*Corresponding Author (Noman.sohail@b-tu.de)

Table S3: Removal Efficiency of TOC across three different operations

Days of operation	Influent mg L^{-1}	Effluent (MBR-1) mg L^{-1}	Removal efficiency MBR-1 (%)	Effluent (MBR-2) mg L^{-1}	Removal efficiency MBR-2 (%)	Effluent (MBR-3) mg L^{-1}	Removal efficiency MBR-3 (%)
1	115.1	22.8	80.2	24	79.1	20.28	82.4
9	124.9	20.6	83.5	21.01	83.2	21.83	82.5
14	82.7	17.4	78.9	17.84	78.4	16.84	79.6
22	103.5	25.8	75	22.05	78.7	27.29	73.6
29	107.8	20.2	81.2	21.15	80.4	20.64	80.8
35	103.6	20	80.6	20.87	79.8	20.98	79.7
42	141.4	19.2	86.5	22.55	84.1	21.69	84.7
51	116.8	24.3	79.2	26.32	77.5	27.74	76.3
60	122.3	22.2	81.8	22.9	81.3	23.39	80.9
72	128.6	17.3	86.6	17.54	86.4	18.04	86.0
81	99.08	18.6	81.8	17.64	82.2	18.87	81.0
93	144.4	19.2	86.6	19.34	86.6	20.56	85.8
103	62.8	9.5	84.9	10.1	83.9	10.2	83.8
Average	112	19.8	82	20.3	81.7	20.6	81.3

Table S4: Removal Efficiency of Ammonia across three different operations

Days of operation	Influent mg L^{-1}	Effluent (MBR-1) mg L^{-1}	Removal efficiency MBR-1 (%)	Effluent (MBR-2) mg L^{-1}	Removal efficiency MBR-2 (%)	Effluent (MBR-3) mg L^{-1}	Removal efficiency MBR-3 (%)
1	129.67	2.11	98	5.73	96	7.75	94
9	110.7	0.85	99	13.26	88	13.22	88
14	216.1	25.6	88	29.58	86	46.76	78
22	113.8	23.92	79	26.48	77	23.22	80
29	138.45	0.89	99	1.78	99	2.15	98
35	131.71	27.82	79	23.17	83	28.1	79
42	133.72	26.24	80	23.39	83	29.42	78
51	133.72	26.24	80	23.39	83	29.42	78
60	94.74	20.88	78	20.66	78	18.66	80
72	105.77	12.99	88	12.77	88	15.64	85
81	105.76	3.05	97	6.06	94	2.37	98
93	95.81	0.4	100	0.48	99	0.68	99
103	101.29	0.4	100	0.47	100	9.99	90
Average	123.94	13.18	90	14.4	88.6	17.5	86.6

*Corresponding Author (Noman.sohail@b-tu.de)
